# Can We Talk About Fetal Consciousness?

**DOI:** 10.1111/apa.70520

**Published:** 2026-03-23

**Authors:** Nadja Reissland

**Affiliations:** ^1^ Department of Psychology Durham University Durham UK

## Abstract

**Aim:**

This mini‐review examines research on fetal consciousness, focusing on whether it can be empirically assessed or remains primarily a philosophical construct.

**Methods:**

A review of theoretical frameworks was conducted. Behavioural evidence from studies of prenatal sensory responsiveness is evaluated, with particular attention to whether observed behaviours are reflexive or intentional actions.

**Results:**

Research in this area is frequently inconsistent. Theoretical models differ in how they conceptualise consciousness and in pinpointing when it may first emerge. Some frameworks propose that consciousness requires the integration of three key components: awareness, meaning the capacity to register sensory input; cognition, defined as the ability to acquire, organise and utilise information; and volition, in terms of intentional, goal‐directed behaviour. Empirical findings from studies of self‐directed fetal movements, behavioural responses to external stimuli, and systematic changes in fetal facial expressions following specific sensory inputs all point towards the *presence of elements of conscious states* rather than the alternative explanation of reflexive activity. These early capacities appear to be observable during prenatal development.

**Conclusion:**

Uncertainty remains regarding the extent to which fetal behaviours evidence full conscious experience. Advancing the field requires controlled experimental research and advanced technology to understand consciousness as it first emerges.

## Summary

- The study is needed because existing theories and empirical evidence on fetal consciousness are inconsistent and require clearer integration
- It found that certain fetal behaviours, including sensory responses and self‐directed actions, may indicate early elements of awareness, cognition and volition rather than simple reflexes.
- These findings suggest the need for more rigorous research and may influence clinical practice in fetal monitoring, pain management and ethical decision‐making.

**Abbreviations**
fEEG: fetal electroencephalography
fMEG: fetal magnetoencephalography
GNW: Global Neuronal Workspace
IIT: Integrated Information Theory
SC: superior colliculus

## 1 Introduction

The question of consciousness, what it is, how it arises and when it begins, has long captivated philosophers, neuroscientists and psychologists alike. Currently, there is an exponential increase in publications referencing ‘fetal consciousness’. The discussion of fetal consciousness has been based on advances in developmental neuroscience, neuroimaging and 4D ultrasound imaging, facilitating the study of prenatal behaviours. Results of such studies have prompted increasing interest in whether and how human consciousness might emerge before birth. This paper summarises some empirical findings in relation to research on fetal touch, sound, vision and taste.

Definitions of human consciousness vary widely across disciplines. One general definition refers to the state of being able to perceive one's surroundings, with thoughts and emotions influencing this percept. Some philosophers, suggest that consciousness cannot be reduced to physical processes and must be understood through introspective or dualist frameworks [1]. Meanwhile, neuroscientists, including for example, Waschke et al. [2], focus on measurable correlates like behavioural responsiveness and neural activation patterns. When applied to fetal studies, these definitions become even more complex. They raise questions of whether consciousness requires emotion, memory or volition, and whether such capacities can be assessed in utero.

Accumulating evidence suggests that foetuses exhibit a range of complex behaviours, which are reactive rather than reflexive. These include self‐directed and environment‐directed movements and specific responses to stimuli that indicate elementary learning. These findings suggest the presence of elements of consciousness, with the caveat of one essential aspect of consciousness, namely reflective capacity, which is evidently not possible at the fetal stage. However, given that it is widely agreed that neonates are conscious [3] without being able to reflect this requirement for a definition of consciousness might not be necessary. Evidence for the potential of prenatal consciousness comes from neuroscientists, who argue that consciousness requires a functioning thalamocortical system which develops from 26 weeks of gestation. However, this is not a failsafe factor in determining fetal consciousness since the timing of a functioning thalamocortical system is disputed with some suggesting that it is functional as early as 25 weeks gestation and others arguing that it is only functional when the infant is around 1 year of age [4]. Nevertheless, prenatal research challenges traditional views of a passive fetus and invites a re‐examination of when functional aspects of consciousness emerge and how conscious states develop over time.

Philosophical ideas of consciousness are especially difficult to apply to prenatal studies. Some philosophers suggest consciousness cannot be reduced to physical processes and must be understood through introspective or dualist frameworks [1]. Arguably, such frameworks could not be applied to the fetus in the womb. Others advocate for physically based models, such as the Global Neuronal Workspace (GNW) [5] and Integrated Information Theory (IIT) [6]. These attempt to quantify consciousness through neural integration and information flow. In this paper, I discuss a limited model using evidence of observable behaviours [7], rather than philosophical definitions of consciousness. This model distinguishes between behaviours, which include elements of consciousness, and those that are merely reflexive.

## 2 Theoretical Frameworks

Using the GNW model, conscious experience emerges when information becomes ‘globally available’ across the brain. This occurs through a network of long‐range neurons. It is hypothesized that this ‘global broadcast’ integrates information from specialised modules for vision, memory and touch. Le Neindre et al. [8], argued in relation to visuomotor processing in the superior colliculus (SC) that multisensory convergence in the SC enhances the detectability and localization of relevant stimuli, particularly when the unimodal signal is weak. According Angelaki et al. [9], brain‐wide recordings using Neuropixels probes reveal that motor‐related and behavioural‐state signals are represented across nearly all regions of the mouse brain, including sensory, motor and subcortical areas. Specifically, Angelaki et al. [9] report that stimulus‐evoked responses in visual areas, first appear in classical visual regions and subsequently propagate to the whole brain, integrating sensory input with ongoing behavioural states and decision‐related signals. In terms of fetal brain development, it is possible that behaviours stimulated in one region propagate to others. This suggests that conscious behaviours could be evident despite immature brain structures. The idea, that not only cortical but also brainstem functions, which develop earlier in gestation than cortical functions [10] play a part in the development of consciousness is also supported by Merker [11] who argued that the brainstem function is essential for consciousness mechanisms as evidenced by goal‐directed animal behaviour observed even after they had their cortex experimentally removed.

Theorists arguing for the GNW contend that consciousness develops from information globally distributed through neural networks, allowing for an integration of information garnered by various cognitive and evaluative systems [12]. Some claim that because of the requirement of a mature prefrontal cortex and thalamocortical connectivity [13], which is not fully developed until late in the third trimester of pregnancy, forms of consciousness can only arise, in a rudimentary form late in pregnancy. However, mouse models show that weak signals can be integrated and amplified [8]. This suggests that behavioural evidence for potentially conscious states could be present sooner. Research using fetal electroencephalography (fEEG) [14], fetal magnetoencephalography (fMEG) [15] and functional Magnetic Resonance Imaging (MRI) [16] have shown complex brain responses to auditory and tactile stimuli. These may reflect early network coordination and hence the developmental progression towards conscious states in the fetus.

Integrated Information Theory (IIT) proposes that consciousness corresponds to the amount of integrated information generated by a system [6]. IIT focuses on causal relationships that can be mathematically modelled. This makes it theoretically applicable to fetal stages. Similarly, Veit [17] argued for conscious abilities in snails by evidencing a minimal sense of ‘evaluative experience’ of *Pterotracheoidea*, which is capable of high‐resolution vision, and has been shown to hunt prey.

Neural Darwinism [18] suggests that consciousness emerges through the selection of neural connections formed by sensory‐motor prenatal experiences. This developmental perspective is supported by fetal studies showing that sensory experiences, including olfaction, taste, touch and sound, shape neural connectivity in the respective sensory pathways as well as in integrative neural networks. For example, exposure to the maternal voice [19] or flavours in amniotic fluid [20] can influence postnatal preferences, indicating that neural selection processes are active before birth. Similarly, the fetal‐neonatal consciousness theory [21, 22] proposes that consciousness emerges in the late fetal period as thalamocortical circuits mature. Their model integrates developmental neuroscience with GNW principles, suggesting that ‘simple awareness’, what I have called an element of consciousness, based on observations of fetal eye opening, may be present in the final weeks of gestation. Trevarthen et al. [23] argue that consciousness is rooted in emotional and bodily states. This perspective focuses on interoception and affective regulation. Foetuses may possess an elementary non‐reflective form of consciousness based on bodily sensations. This is supported by fetal reactions to maternal mental health [24] and flavours [25].

To summarise, because biophysical models require a relatively mature brain, including a mature cortex, which can be only found in later gestation, researchers subscribing to biophysical models, tend to claim that early gestational foetuses cannot be conscious. However, others argue that there are neuronal connections which temporarily exist and then disappear as the fetus matures [26] and therefore make claims of the basis of consciousness earlier in gestation possible. Regarding evidence of fetal consciousness experimental studies investigating behaviour and brain development in the human fetus have provided some data suggesting the potential of consciousness before birth. Based on a behavioural definition of consciousness, there are an increasing number of studies demonstrating that foetuses exhibit behaviours and neural responses that may reflect at a minimum, evidence of elements necessary for ‘conscious’ states. In the context of fetal development, the GNW model suggests that consciousness emerges when information is globally broadcast across brain regions, a process that may not be fully operational until late in gestation. Based on a behavioural model of consciousness the current mini narrative review discusses main points of the current literature on fetal consciousness, citing experimental work of fetal touch, taste, vision and sound.

## 3 Research on Fetal Learning and Memory

Evidence of learning and memory indicates the first steps towards conscious reactions. Fetal reactions to auditory stimulation have been widely studied. Research indicates that foetuses detect and respond to external sounds including voices and music. Studies using heart rate monitoring and movement analysis using actograms have shown that foetuses habituate to repeated auditory stimulation. This indicates short‐term memory and sensory discrimination [27, 28, 29, 30]. Memory of prenatal sound stimulation is indicated by research, which reported that newborns showed a preference for stories or songs heard prenatally [31], suggesting long‐term memory formation during fetal life. Other research using 4D ultrasound indicates that foetuses make mouth movements necessary for specific sounds [32]. In this study one group of foetuses was exposed experimentally to a specific sound (‘ma’) and another group did not hear this sound. Two different types of mouth movements, either mouth stretch or lip pucker, were coded frame by frame from ultrasound recordings at 32 and 36 gestational weeks. Fetuses in the sound group, hearing ‘ma’ performed the mouth stretch movement significantly more frequently than foetuses in the no‐sound group. In contrast the second mouth movement coded, namely lip puckering did not increase when foetuses heard the sound ‘ma’. These results support a conclusion that a specific sound stimulus is associated with an increase in a specific, rather than general, mouth movement. These findings align with theories that emphasise information integration and sensory awareness as elements of consciousness. The ability to encode, retain and respond to auditory stimuli implies a level of cognitive processing that goes beyond mere reflex behaviour.

The fact that fetal memory persists into the postnatal period for flavour stimuli experienced prenatally has been demonstrated in research coding fetal and neonatal facial expressions. Using 4D ultrasound imaging of facial expressions at 32‐ and 36‐weeks' gestation and video recordings in the first month after birth, were coded. Foetuses were stimulated with either carrot flavour (non‐bitter) or kale flavour (bitter) via maternal ingestion of calorie‐controlled capsules of carrot or kale powder. The control group was not stimulated with any specific flavour. Foetuses stimulatd with carrot flavour in the womb showed ‘lip‐corner puller’ and ‘laughter‐face gestalt’ more frequently, compared to foetuses stimulated with kale flavour showing more ‘upper‐lip raiser’, ‘lower‐lip depressor’, ‘lip stretch’, ‘lip presser’ and ‘cry‐face gestalt’. Furthermore, foetuses stimulated with the bitter flavour showed a decrease in cry face gestalt expression from being stimulated at 32 weeks gestation to being stimulated at 36 weeks gestation, indicating that this was not a reflex activity [25]. Additionally, after birth, these neonates of mean age = 3.06 weeks showed a decreased frequency of a cry‐face, and an increased frequency of a laughter‐face gestalt in response to the odour stimulus experienced prenatally, regardless of whether the taste was bitter or non‐bitter [20]. These results indicate memory for repeated chemosensory experience in utero. Arguably, these findings suggest that foetuses show specific reactions when exposed to prenatal experiences, a process potentially involving emotional and cognitive components. Such findings support theories of consciousness, which posit that bodily and emotional states form the basis of awareness. The ability to distinguish between ‘pleasant’ and ‘unpleasant’ stimuli, and to remember them, may reflect early forms of subjective experience.

## 4 Touch and Movement in Singleton and Twin Pregnancies

Prenatal tactile behaviours help delineate the developing self in relation to the other, where the ‘other’ may be a co‐twin in pregnancies of multiples or the uterine environment [33]. A key indicator of emerging conscious behaviour is the sequence and arguably intentionality of self‐directed movements (e.g., [34]). For example, results of anticipatory action developing in foetuses observed from 24 to 36 weeks gestation showed a significant increase in the proportion of anticipatory opening of the mouth before touch increasing, by around 8% with each week of gestational age, and suggesting purposive action rather than reflexive movement [35].

Regarding volition, based on Yamada et al.'s [36] robot model, prenatal stimulation through touch could provide the foundations for cortical learning of body representations. These body representations are hypothesized to provide foundations for postnatal visual‐ somatosensory integration [36, 37]. Studies examining fine‐grained fetal behaviours using 4D imaging show that foetuses engage in apparently purposeful behaviours such as hand‐to‐mouth movements, facial touching, and, in case of twin pregnancies, even differential tactile interactions in utero between their own body and their twin's body [33, 35, 38]. These behaviours suggest a degree of volitional control, challenging the notion that fetal movement is purely reflexive.

One argument for developmental stages of consciousness is that both singletons and twin touch behaviours change depending on maternal stress and depression. Specifically, studies on twin pregnancies [33, 38] indicate that foetuses use differential patterns of touch towards themselves and their co‐twin, depending on maternal stress and depression. In a study examining singleton and twin fetal touch behaviours we found that maternal depressive mood significantly increased self‐touch of both singleton and twin foetuses of 27 weeks gestational age. In twin foetuses we found that maternal mental health affected touch behaviours differently in that maternal *stress* significantly decreased other twin touch. In contrast, maternal *depressive* mood significantly increased other twin touch [24]. This research suggests that foetuses seem to be able to differentiate self from other, an ability which is included in some definitions of consciousness and therefore evidences a potential element necessary for conscious abilities. Furthermore, developmental progression towards more specific behaviours across gestation, supports the idea of a development of intentional actions, a key component in many models of consciousness.

Another test potentially indicating intentional actions relates to fetal head turns at 33 weeks gestational age to light stimulation changing depending on maternal mental health [39]. Maternal increased depression score reduced fetal head turn frequency to light stimulation significantly (*p* < 0.001). In contrast, mothers with increased anxiety had foetuses who showed significantly more head turns in reaction to light stimulation (*p* < 0.004). Hence, foetuses seemed to change their behaviour depending on how anxious or depressed their mothers were.

Regarding volition, fetal touch behaviours could provide the foundations for cortical learning of body representations [33, 35]. If we agree that foetuses develop actions such as inserting their fingers into their mouths [35] and in twins showing differential touch behaviours of self and co‐ twin depending on maternal mental state [33], then we might argue that foetuses show some indications of volition. Other studies show that neonates remember flavours they were exposed to prenatally (e.g., [25]). Specifically, foetuses who were stimulated at 32 weeks to a bitter flavour (kale) showed facial expressions resembling disgust which were *reduced* if they were stimulated again at 36 weeks gestation indicating fetal memory or possibly reactions to familiarity of the bitter flavour. This interpretation has been reinforced by a follow up study with 1 months old neonates, who were exposed either to kale or carrot flavour prenatally and who in the neonatal stage reacted differently to the smell experienced prenatally and the smell not experienced prenatally. The neonates reacted more positively in terms of facial expressions and bodily movements to the smell they had experienced prenatally and more negatively to the smell not experienced prenatally irrespective of whether it was the carrot or kale smell. Although we cannot show that foetuses reflect on their own mental or physical state, they are demonstrably influenced as evidenced by changes in their behavioural profile by external and internal stimulation. Hence, depending on the definition of consciousness, we would be able to talk about elements of ‘conscious’ states being evidenced in fetal behaviours.

## 5 Conclusion

Current evidence suggests that consciousness is not a binary, ‘all‐or‐nothing’ state. Instead, it exists on a developmental continuum of increasingly complex neuronal interactions [40]. We must therefore stop asking *if* the fetus is conscious and start asking which *elements of consciousness are evident* at different stages of fetal development.

Significant challenges remain before fetal consciousness can be unequivocally asserted. The first is conceptual; the definition of consciousness is still debated even in adults. This makes establishing clear criteria for the fetus difficult given that, as Tononi et al. [41] argue, ‘we need to account objectively for its subjective presence and properties’. The second challenge is technical. Ethical constraints limit invasive procedures, and non‐invasive techniques often suffer from low resolution. Consequently, we must rely on an inference (e.g., Le Neindre et al.; Ehret and Romand; Nieder) [42, 43, 44].

The fundamental challenge is differentiating between reflexive movements and genuinely intentional actions. Although many fetal behaviours may *appear* purposeful, establishing true intentionality remains difficult. Nonetheless, emerging research on multisensory fetal experience indicates that, particularly in the late third trimester, the fetus exhibits a behavioural repertoire that aligns closely with the foundational elements of conscious action.

## Author Contributions

**Nadja Reissland:** conceptualization, investigation, writing – review and editing.

## Funding

The author has nothing to report.

## Conflicts of Interest

The author declares no conflicts of interest.

## Data Availability

Data sharing not applicable to this article as no datasets were generated or analysed during the current study.
